# Dental caries shallow-layer microbe harvest and data display method shows taxa pre-harvest spatial positions, absolute and relative abundance and diversity related to lesion pulpal depth

**DOI:** 10.1080/20002297.2025.2593129

**Published:** 2025-12-04

**Authors:** Rella P. Christensen, Brad J. Ploeger, Kaesy R. Barker, Wyatt R. Hume, Brenda Heaton

**Affiliations:** a TRAC Research, Provo, Utah; b University of Utah, School of Dentistry, Salt Lake City, Utah

**Keywords:** Dental caries/microbiology, taxonomy/microbial, microdissection, specimen handling/methods, microdissection, shallow layer, pre-harvest microbe positions

## Abstract

**Background:**

Molecular identification of dental caries microbes is advancing rapidly, yet sampling methods remain outdated and imprecise.

**Objective:**

To refine microbe sample harvesting and preserve taxa pre-harvest spatial positions relative to lesion pulpal depth.

**Methods:**

Refinements included a sterile zone surrounding the dissection site and emphasize asepsis, surgical microscope magnification and lighting, and micro-surgical techniques. Retention of taxa pre-harvest spatial positions relative to lesion pulpal depth used shallow-layered dissection (mean 6 mg/layer, SD 2.65 mg), where each layer became a separate sample for molecular identification before sequential reassembly in a layered lesion diagram. To evaluate the method’s robustness, 14 lesions varying in severity and type from private dental practices were dissected using the method (7 pit and fissure and 7 facial lesions; 4 untreated, 3 treated).

**Results:**

Pre-harvest taxa detail, not possible previously, showed taxa location, abundance and diversity relative to lesion pulpal depth, as well as absolute abundance per milligram and taxa transitions and fluctuations from superficial through the deepest dissected layers. The method provides these data regardless of lesion type, stage, or complexity, whether untreated or treated.

**Conclusions:**

This method provides new details and perspectives on dental caries taxa that could help develop diagnostic instruments and treatments to halt dental caries progression.

## Introduction

Molecular methods are increasing the sensitivity of taxa identification within dental caries lesions, but taxa harvest methods providing the crucial samples are not keeping pace, and are described as ‘crude’ [1,2]. This disparity requires attention. Conspicuous problems with current harvest methods include: inadequate isolation surrounding the harvest site, which hinders the harvest of contaminant-free samples; the removal of lesion contents in arbitrary clumps followed by homogenization, which obliterates pre-harvest taxa spatial positions relative to the pulpal depth; and disregard of available technology that enhances visual access, critical for precise sample selection, dissection and harvest [2–11].

To address these problems and align sample harvest sensitivity more closely with molecular identification sensitivity, we propose a new dental caries harvest method. This method incorporates a sterile zone encircling the lesion, uses surgical microscope magnification and lighting to maximize vision, and employs micro-surgical shallow-layer dissection. Each dissected layer is harvested separately, and the taxa identified per layer are then used to construct a layered lesion diagram, which shows for the first time, the taxa present in their pre-harvest positions relative to lesion depth in the pulpal direction, and their pre-harvest transitions and fluctuations in diversity and in both absolute and relative abundance throughout the lesion. The purpose of this report is to present a full description of the new method and to provide example layered lesion diagrams displaying the new information made possible using the method *in situ* in lesions of varying type, stage and complexity, including both untreated and operatively and non-operatively treated lesions.

### Materials and methods

The following method was developed to combine surgical microscopy, micro-dissection, layered sampling, and a new data display design that presents a layered diagram of the pre-harvest location of a lesion’s internal taxa relative to its pulpal depth. This section describes the design of the aseptic operating area, the procedural steps for shallow-layer dissection and sample collection, and the protocol used for microbial processing and data visualization. Each component of the method was developed to provide sample integrity, minimize contamination, and allow reconstruction of the pre-harvest microbial distribution relative to the pulpal depth of the lesion.

### Aseptic operating area and contents

**Figure 1.**
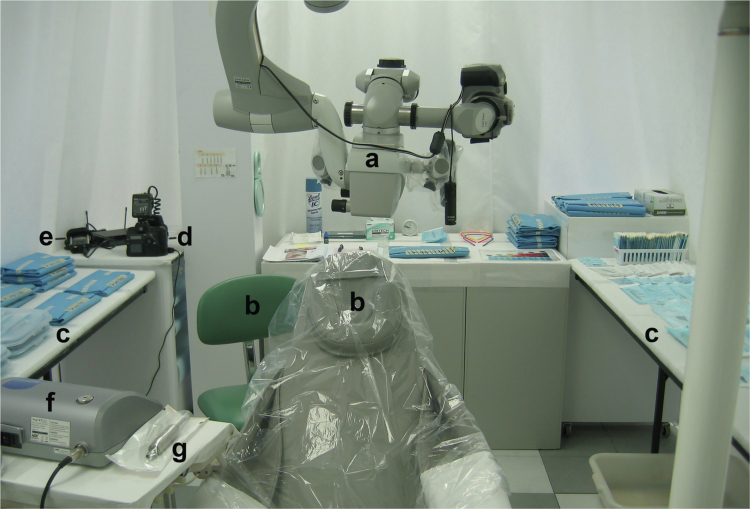
Appearance of the operating setup within the curtain isolated area before dissection procedures. (a) Ceiling-mounted surgical microscope. (b) Operator and subject chairs. (c) Portable surfaces (barrier covered) with sterile packaged instruments and supplies arranged in order of use. (d) Fuji SLR camera. (e) Audio recording equipment. (f) Foot-operated surgical electric control unit that powers the electric motor (g).

A vacuum evacuated microbiology laboratory was floor-to-ceiling curtain-partitioned to form an isolated space of 14 x 6 x 8 feet that included a 2 × 4 foot downdraft HEPA air filtration unit (Fantom, Gordon Cleanroom Products) ceiling-mounted over the oral cavity operating area and confirmed at 1.2 air exchanges per minute at high setting (Hot Wire Thermoanemometer WD20250-16, Digi-Sense). Items within the operating area included: a dental subject chair with attached light; a clinician chair; a ceiling-mounted surgical microscope (PROergo, Zeiss); a surgical electric control unit with 20 handpieces and 20 attachments (Surgic XT Plus, NSK) to aseptically power micro-size cutting burs at low rpm using no compressed air or water; a hand-held SLR camera with a close-up lens (S-1 Pro, Fuji with 105 mm lens, Nikon) to produce high-quality close-up digital photographs of lesion appearance during dissection; and an audio recording system (Tascam DR-44WL, Teac) to record real-time observations during dissection (see Figure 1).

Equipment and instruments outside the isolated area included: two certified anaerobe chambers designated as chamber #1 (AS−580, Anaerobe Systems) containing a certified microbalance (MSA6.6SOTRDM, Sartorius) and deionizer (STAT-FAN 0-dc/YIB01-OUR, Sartorius) for weighing samples; chamber #2 (Whitley MG500, Microbiology International) for dividing each layer sample into two aliquots used for culture and Sanger sequencing and ultra-low temperature storage; a portable incubator (Incufridge RS-IF-202, Revolutionary Science) for warming sterile resin dam material; a steam sterilizer (PSS11-AA-MSSD, Primus); a HEPA air filtered cleanroom with a certified biologic safety cabinet (NU-543-600, Nuaire); incubators; refrigerators; and ultra-low temperature freezers.

### Aseptic shallow-layer dissection procedure

Steps below are ordered as performed by a team wearing protective coverings appropriate for their assigned functions.

**Figure 2.**
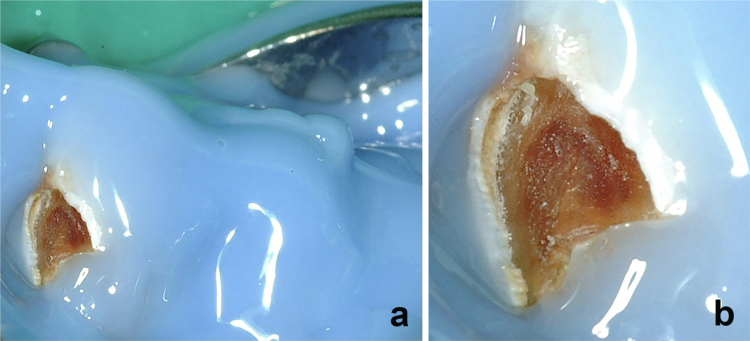
Sterile resin dam dissection site isolation. (a) Sterile resin dam aseptic application in progress to cover the disinfected elastic dam and metal clamps. (b) Close-up of the completed resin dam applied to create a sterile zone surrounding the lesion and prevent ingress of oral fluids.

**Figure 3.**
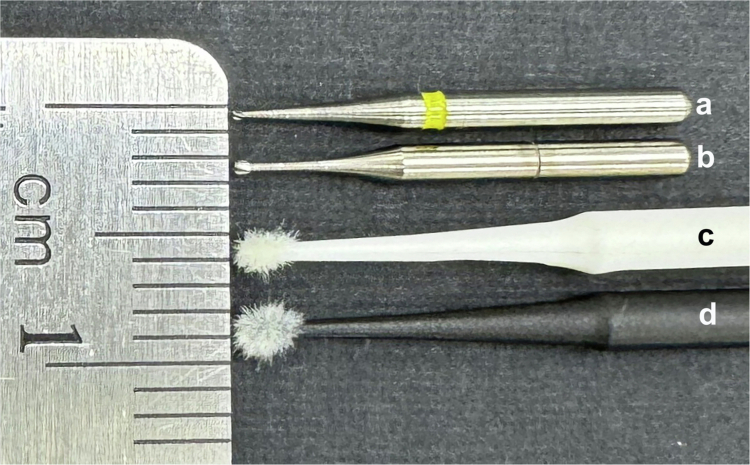
Micro-dissection burs and micro-collection brushes compared to a millimeter scale. (a) ⅛ round carbide bur. (b) ¼ round carbide bur. (c) Magic-Brush Extra Small. (d) Multi-Brush Extra Small.

**Figure 4.**
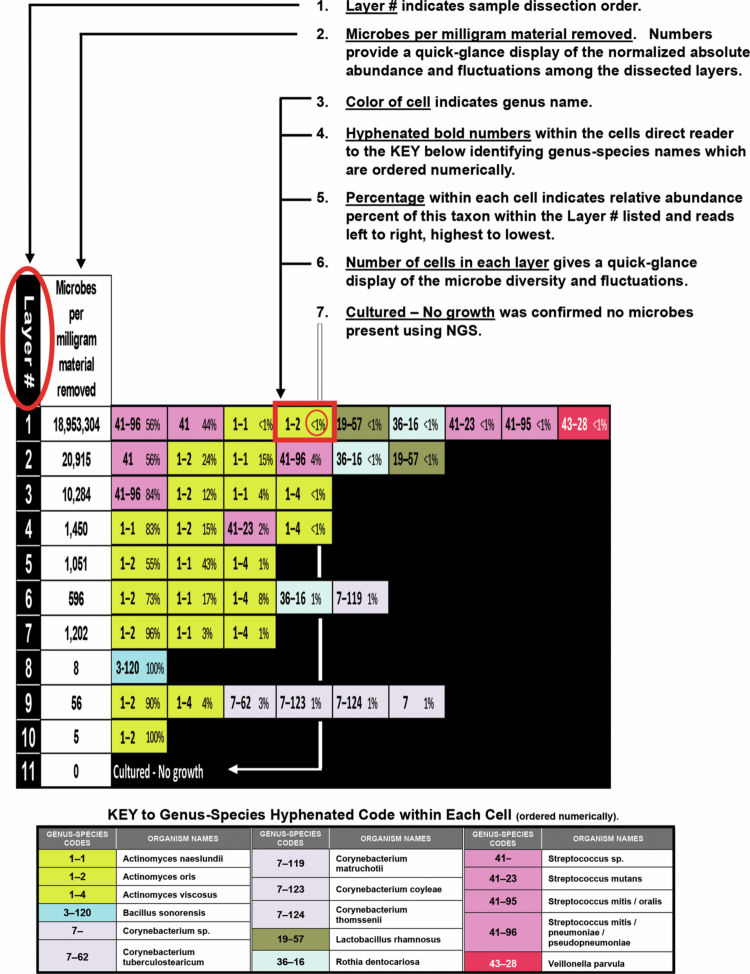
Directions for reading layered lesion diagrams. (Lesion diagram generated from culture with Sanger sequencing.)

**Figure 5.**
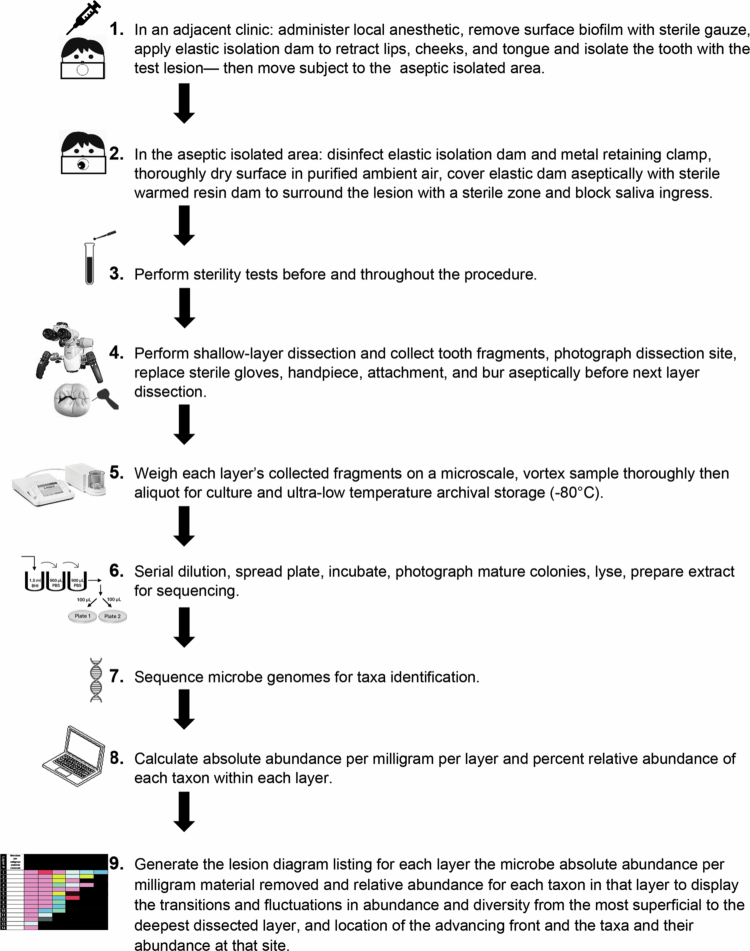
Summary of steps in the new shallow-layer harvest and data display method.

**Figure 6.**
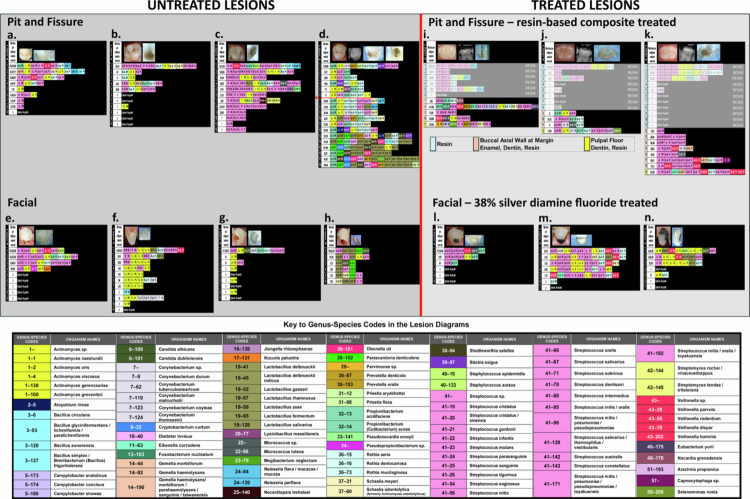
Layered lesion diagrams and Key for 14 lesions varying in type, stage and complexity, untreated and treated, selected from private dental practices to test the new method’s scope and potential. (a) A moderate lesion containing notably high numbers of microbes per milligram of material removed. (b and c) Two moderate lesions in different subjects that appear similar clinically, but vary substantially internally in microbe abundance and diversity. (d) An extensive pit and fissure lesion intersected by a proximal lesion between layers 6 and 7 (red arrow) illustrating taxa transitions and fluctuations as it progresses pulpally. (e–h) Four extensive untreated facial lesions showing variations in abundance and diversity, with lesion (h) also showing a shift in taxa predominance from *Streptococcus* in layer 1 to *Lactobacillus* in layers 2−6. (i–k) Three occlusal resin-based composite restorations dissected ~1.5 years after placement, showing high taxa abundance and diversity under the restorations, and at and below the resin‒dentin interface. (l–n) Three extensive 38% silver diamine fluoride treated facial lesions showing residual taxa abundance and diversity below the lesion surface following the final SDF treatment at 10 days (l), 12 days (m), and 90 days (n).

1. **Subject pre-dissection preparation:** In an adjacent outpatient dental clinic, the subject received local anesthetic, had surface biofilm removed using sterile gauze and sterile gloves, and placement of an elastic dam to provide retraction of lips, cheeks, and tongue and isolate the tooth containing the test lesion.
2. **Elastic dam disinfection:** Within the isolated area, where air purification had been operated at high setting 48 h before and throughout treatment, the elastic isolation dam and its metal retaining clamps were disinfected with 2 ml 95% ethyl alcohol (Everclear Grain Alcohol, Luxco) pipetted aseptically and separately just before use onto five separate sterile gauze sponges, used and discarded one at a time, while surfaces were allowed to dry between each sponge used in the purified ambient air (total wet contact time ≥5 min). NOTE: We have data that indicate non-denatured USP grade ethyl alcohol at 89−95% v/v produces a more reliable broad-spectrum kill in short contact times (30−60 s) than the commonly available 70% v/v denatured ethyl alcohol or isopropyl alcohol.
3. **Dissection site sterile isolation:** Sterilized warmed (37°C) resin dam material (Dental Dam, Den-Mat) was syringe applied and immediately light-polymerized over small contiguous sections of the thoroughly dry elastic dam, metal clamps and intact tooth surfaces where it formed a fragile seal that ultimately surrounded the dissection site to provide a sterile zone surrounding the operating site and prevent saliva ingress (see Figure 2). NOTE: Foregoing steps 2 and 3 were used when culture and Sanger sequencing were performed. If culture-free NGS is used, the disinfection described by Widmer et al., should be considered [12].
4. **Sterility monitoring:** Samples of all sterilized items were monitored for sterility before and throughout the procedure to verify maintenance of sterile conditions. (For monitoring results, see Supplementary Table S1: Sterility control results for the 14 lesions in Figure 6).
5. **Electric motor aseptic assembling:** Immediately before tooth dissection, the surgical electric control unit connected to the barrier-covered electric motor received, sequentially and aseptically, a sterile handpiece, a sterile attachment, and an unused sterilized ⅛ or ¼ round carbide bur, long or short shanks (Brasseler).
6. **Shallow-layer dissection procedure:** Lesion dissection began when the surgical electric control unit, set at 1,000 rpm (2,000 for enamel), was foot activated and the bur applied in intermittent, light-touch slow stroking motions on the lesion or restorative material surface with gentle minimal surface contact, allowing the miniature, sharp new blades to dislodge fine fragments in a controlled manner. The dissection was restricted to the lesion core, excluding the edges. Dissection of the resin-based composite-treated lesions in posterior teeth started at the central fissure of the restoration and moved in shallow layers to the buccal resin-tooth interface, then moved in shallow layers apically to the pulpal floor of the tooth preparation (the lingual margins remained undisturbed and were covered with sterile resin dam material). Bur use was stopped repeatedly after short intervals, and the fragments were collected to maintain visual access, prevent fragment scattering, and prevent heat buildup. The mean fragment weight per dissected layer was: mean 6.00 ± 2.65 mg; range 0.60−9.70 mg. The amount of material removed per sample was decreased when close to the pulp chamber.
7. **Sample collection:** Dislodged fragments were collected onto filaments of pre-weighed sterilized brushes (Magic-Brush Extra Small and/or Multi-Brush Extra Small, Denbur), which were dampened in sterile brain heart infusion (BHI) broth immediately before use to facilitate pick-up (see Figure 3). Once filled, each brush was deposited immediately and aseptically into a previously letter-coded pre-weighed sterile screw top vial (Nunc^TM^ Cryotube Vial, Thermo Fisher Scientific) containing 1.5 ml of sterile reduced BHI broth. Consecutive letters were used to identify the dissected layer order. The vial was brought adjacent to the dissection site, uncapped just before entry of each brush, recapped immediately after receiving each brush, and moved away until needed again for loading of the next brush. Each vial was allowed to contain a maximum of four brushes. Dissection of each new layer received new sterile items including gloves, unused bur, handpiece and attachment, pre-weighed collection brushes, and pre-weighed vial containing BHI medium.
8. **Collected sample processing:** Each letter-coded dissected layer corresponded to one sample for culture, molecular sequencing and taxa identification, and was processed as follows: Immediately after each vial was loaded, it was transported by a runner to anaerobe chamber #1 and weighed, then taken to anaerobe chamber #2, where it was vortexed and separated into two aliquots. One aliquot received glycerol to 20% (v/v) and was frozen at −20°C for 10−12 h before transfer to an ultralow temperature freezer (−80°C) for archival storage. The second aliquot remaining in the cryotube containing the micro-brushes was retained in anaerobe chamber #2 for serial 1:10 dilution using Phosphate Buffered Saline (PBS, Thermo Fisher Scientific), and 100 µL was plated onto duplicate pre-reduced blood agar plates (Remel Anaerobic (CDC) Blood Agar (RO1036), Thermo Fisher Scientific) and then incubated anaerobically (80%N_2_, 10%CO_2_, 10%H_2_) at 37°C until colonies matured (7−21 days). Mature colonies were digitally photographed, lysed, and the extract was prepared for sending to a commercial laboratory (Charles River Laboratories, Newark, DE) for Sanger sequencing and taxon identification, where the molecular identity of the isolates was determined via proprietary 500 bp 16S rDNA sequencing and analysis methods, AccuGENX-ID, and the identification reference library at Charles River Laboratories. The DNA was isolated via standard alkaline lysis methods, and the extracts were subjected to PCR amplification with standard M13-tailed 16S primers, namely, 0005F (TGGAGAGTTTGATCCTGGCTCAG) and 0527R (GTATTACCGCGGCTGCTGGCAC) with the primer numbering based on the *Escherichia coli* 16S rRNA gene [13]. Sequencing reactions were carried out using manufacturer's recommendations and BigDye^TM^ Terminator v1.1 cycle sequencing kits (Thermo Fisher Scientific, Carlsbad, CA) and run on an ABI 3130XL or ABI 3500XL Genetic Analyzer capillary sequencer (Thermo Fisher Scientific, Carlsbad, CA). Data meeting a Phred score of ≥20 on the internally developed sequencing assessment software were assembled and analyzed via a semiautomated reference method, and sequences were compared to the Accugenix proprietary, validated, reference database for identification. The 16S rRNA gene sequences of the samples were aligned together with those of other closely related reference species and analyzed phylogenetically using proprietary software. Evolutionary distance was calculated on the basis of the Jukes and Cantor model [14] and phylogenetic trees were inferred on the basis of the Saitou and Nei neighbor-joining models [15]. Identifications were made upon consideration of the sequence alignment and the neighbor-joining tree for each isolate.
9. **Photographic documentation:** When tooth fragment collection was completed for a layer, the dissection site was prepared for digital photographing by dampening a new sterilized Multi-Brush or Magic-Brush with sterile BHI broth used to moisten the dissected area to accentuate the surface color and detail in close-up images produced with the hand-held SLR digital camera. The brush used for moistening was discarded. In preparation for dissection of the next layer, all used sterilized items were replaced (see step 7 above for a list of items).
10. **Dissection termination:** The above steps were repeated until two clinicians agreed on the dissection termination based on tissue hardness, appearance and/or pulp chamber proximity.
11. **Post-dissection site restoration:** After dissection completion, the subject was returned to the adjacent dental clinic to receive a restoration placed by the clinic’s dentist to restore the tooth’s form, function and esthetics.
12. **Layered lesion diagram creation:** The final step was to create a layered lesion diagram to communicate taxa identification results, visually and numerically (see Supplementary Figure S1 for serial dilution scheme and CFU/mg calculation), displaying the taxa identified in each consecutive layer dissected. Figure 4 shows an example lesion diagram and directions related to how to read a lesion diagram. Figure 5 summarizes the method steps.

For additional details, see protocols.io at doi.org/10.17504/protocols.io.bp2l6dmdkvqe/v1.

### Clinical lesions to test the robustness of the method

To evaluate the scope and potential of the shallow-layer harvest and display method, it was applied to a diverse group of dental caries lesions. Patients were recruited from dental practices across multiple US states to ensure real-world relevance and independence from the research team. This investigation was approved by the University of Utah Institutional Review Board (IRB #00177483).

Lesions varying in type, stage and complexity, both untreated and treated, were sought. The subject inclusion criteria were: 24 teeth minimum, aged 18−70 years, free of major systemic disease, and willingness to participate in the investigation. The exclusion criteria were: lesions possibly involving endodontic complications, soft tissue lesions in the oral cavity or on the head or neck, reported allergies to materials used in the study, antibiotic or anti-fungal use within 6 months of study participation, pregnancy, high dental anxiety and familial relation to another subject participating in the evaluation.

Signed informed consent was obtained from all the subjects. The selected subjects included both genders, ages 26−64 years, who resided in different regions of the United States (Idaho, Pennsylvania, Tennessee, Utah). Four categories of lesions were selected for inclusion: untreated pit and fissure lesions (*n* = 4), untreated facial lesions (*n* = 4), pit and fissure lesions treated with resin-based composite restorations planned for replacement by the patients and their dentist (*n* = 3), and facial lesions treated with 38% silver diamine fluoride (*n* = 3). Table 1 lists the characteristics of the lesions and subject demographics [16]. Each lesion was dissected following the shallow-layer harvest and data display method as described previously.

**Table 1.** Characteristics of the 14 lesions selected to test the scope and potential of the shallow-layer microbe harvest and data display method and subject demographics.

	Lesion characteristics				Subject demographics											
Lesion designation in Figure 6	Category	Tooth type	Caries Stage*	Caries Activity*	Gender	Age	Race	Education	Employment	Socio-economic Position	Added sugar/low pH beverage use	Fluoride dentifrice use	Percent teeth with visible biofilm	Unstimulated saliva flow	Active caries throughout oral cavity	Caries Risk*
	UNTREATED LESIONS															
a.	Pit & fissure	Premolar, lower second	3 Moderate	Active	M	32	White	High School	Part-time	Low	High	Occasionally	100%	≥1 mL/min	High	High
b.	Pit & fissure	Molar, upper first	3 Moderate	Active	M	64	White	High School	Part-time	Low	High	Occasionally	50%	<1 mL/min	High	High
c.	Pit & fissure	Molar, upper second	3 Moderate	Active	F	28	White	Bachelor's Degree	Full-time	Middle	High	Every Night	30%	≥1 mL/min	High	High
d.	Pit & fissure + mesial	Premolar, upper second	5 Extensive	Active	M	50	White	Bachelor's Degree	Full-time	Middle	High	No	100%	<1 mL/min	High	High
e.	Facial	Premolar, lower first	5 Extensive	Active	F	32	White	Bachelor's Degree	Full-time	Middle	High	Occasionally	100%	<1 mL/min	High	High
f.	Facial	Incisor, upper lateral	5 Extensive	Active	M	26	White	High School	Full-time	Middle	High	No	100%	<1 mL/min	High	High
g.	Facial	Canine, lower	5 Extensive	Active	M	27	White	High School	Full-time	Middle	High	No	100%	<1 mL/min	High	High
h.	Facial	Canine, lower	5 Extensive	Active	M	27	White	High School	Full-time	Middle	High	No	100%	<1 mL/min	High	High

	TREATED LESIONS															
i.	Pit & fissure Resin composite	Molar, lower first	Unable to determine	Unknown, Replaced per subject request to dentist	F	28	White	Bachelor's Degree	Full-time	Middle	High	Yes	7%	≥1 mL/min	High	High
j.	Pit & fissure Resin composite	Molar, lower first	Unable to determine	Unknown, Replaced per subject request to dentist	F	26	White	High School	Full-time	Middle	High	Yes	10%	≥1 mL/min	High	High
k.	Pit & fissure Resin composite	Molar, lower second	Unable to determine	Unknown, Replaced per subject request to dentist	F	26	White	High School	Full-time	Middle	High	Yes	10%	≥1 mL/min	High	High
l.	Facial 38% SDF	Premolar, lower first	5 Extensive	Active	F	32	White	Bachelor's Degree	Full-time	Middle	High	Occasionally	100%	<1 mL/min	High	High
m.	Facial 38% SDF	Premolar, lower first	5 Extensive	Active	M	36	White	High School (incomplete)	Part-time	Low	High	No	100%	<1 mL/min	High	High
n.	Facial 38% SDF	Incisor, upper lateral	5 Extensive	Active	M	26	White	High School	Full-time	Middle	High	No	100%	<1 mL/min	High	High

* ICCMS^TM^ guide for practitioners and educators^16^.

## Results

Figure 6 displays the 14 layered lesion diagrams that resulted from the use of the shallow-layer harvest and data display method on the four categories of lesions selected for study to test the robustness of the method. Clinical images taken before and after the completion of each dissection, and radiographs pre-dissection of the resin-based composite-treated pit and fissure lesions (6i–k) and a conjoined untreated mesio-occlusal lesion (6d) were included to provide orientation on the clinical aspects of the lesions and the dissections.

The shallow-layer harvest and data display method showed the untreated facial lesions had substantially fewer genera than the pit and fissure lesions (14 compared to 29). The genera detected in the pit and fissure lesions, but not in the facial lesions were *Atopobium, Campylobacter, Cryptobacterium, Dialister, Gemella, Jiangella, Lysinibacillus, Micrococcus, Mogibacterium, Neisseria, Nocardiopsis, Olsenella, Prevotella, Priestia, Pseudonocardia, Shuttleworthia, Slackia* and *Streptomyces*. The predominant genus across the four untreated facial lesions was *Streptococcus*, at 99% in all four lesions. Across the four pit and fissure lesions, *Streptococcus* and *Propionibacterium* were the predominant genera. The resin-based composite and 38% silver diamine fluoride (SDF)-treated lesions showed opposite patterns of taxa diversity and abundance in their deepest dissected layers. The resin-based composite-treated lesions had the greatest taxa diversity and high abundance under the restorations, with *Parvimonas* predominant below one of the restorations (6i), and *Streptococcus* predominant below the other two restorations (6j–k). In contrast, the 38% SDF-treated lesions had the least diversity and low abundance in the deepest layers, with *Streptococcus* predominant in all three lesions. Neither treatment substantially lowered abundance or diversity in the superficial layer.

All three resin-based composite lesion diagrams showed high levels of taxa absolute abundance and diversity under the resin restorations, on the pulpal floor of the cavity preparation. The shallow-layer harvest and data display method allowed reporting of all the identified taxa and indicated where those taxa were located relative to pulpal depth, their absolute abundance per milligram of material removed, and the relative abundance of each taxon within each dissected layer under the resin composite restorations.

**Figure 7.**
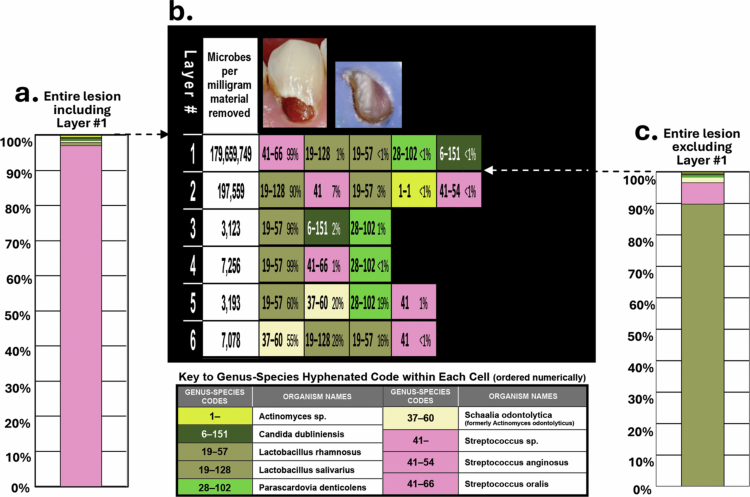
An example of an untreated facial lesion (Figure 6h) illustrates the additional information possible on lesion internal abundance and diversity when the shallow-layer harvest and data display method is used. The lesion diagram (b) and 100% bars (a) and (c) demonstrate a shift in taxa predominance from *Streptococcus* in layer 1 to *Lactobacillus* in layers 2−6 that would not be evident using other harvest methods that remove clumps of material and/or pool or homogenize multiple harvests from the same lesion. The extremely high absolute abundance per milligram of *Streptococcus* in layer 1 prevents recognition of the predominance of *Lactobacillus* in the lesion body and advancing front.

Specific lesions in Figure 6 that demonstrated detection and display of subtle internal details within *in situ* dental caries lesions were: (1) Lesion 6a showed a negligible appearing lesion that contained exceptionally high numbers of microbes per milligram throughout the lesion; (2) Lesion 6d showed the taxa involved and their location relative to pulpal depth of the shifts and transitions within a conjoint mesio-occlusal lesion where the point of intersection of the two lesions showed an abrupt 3 log_10_ increase in taxa abundance between layers 6 and 7 (95% Cl: 2.62, 4.68), and by layer 13, a substantial shift in taxa that added 33% new genera and 43% new species to the overall lesion content; (3) Lesion 6h revealed a shift in genera predominance, with *Streptococcus* predominant in layer 1, but several *Lactobacillus* species predominant in layers 2−6. Figure 7 illustrates this shift.

All the above considered together demonstrated the method’s potential to show details within a diverse group of dental caries lesions by displaying: (1) diversity per layer; (2) absolute abundance per milligram of material removed per layer and relative abundance of each taxon within each layer; (3) taxa transitions and fluctuations in diversity and abundance from the superficial through the deepest dissected layers; and (4) taxa identity and pre-harvest abundance (both relative and absolute) within the deepest layers dissected at each lesion’s advancing front. The 14 diagrams also demonstrated the method’s use in lesions that varied in type, stage and complexity, whether untreated or treated operatively or non-operatively.

## Discussion

The intent of this report is to describe a new method designed to harvest microbes from dental caries lesions aseptically, precisely, and in a manner that preserves the pre-harvest positions of the taxa relative to the pulpal depth of the lesion. The aim is to more closely align the quality of the harvested samples with the sensitivity of molecular identification methods now capable of detecting picogram quantities of target DNA [17]. The most important features gained by this method are: (1) improved assurance that all taxa identified originated from the target site through the use of strict asepsis, including a sterile zone surrounding the dissection site; (2) precision in the selection, dissection, and harvest to allow repeated aseptic acquisition of optimum-quality sample material through the use of surgical microscope magnification, lighting and micro-surgical instruments; (3) a new visual and numerical representation of pre-harvest taxa information in a layered lesion diagram displaying clearly the layer where specific taxa are located relative to the lesion’s pulpal depth and the transitions and fluctuations in diversity and abundance (absolute and relative) from the most superficial to the deepest dissected layers. This level of detail on the internal organization of the pre-harvest taxa within dental caries lesions has not been reported previously.

Researchers and clinicians have harvested and studied the contents of dental caries lesions for more than 100 years [18–23], but surprisingly, very few changes in the methods and implements used have been reported. Historically, and continuously to the present, a spoon excavator hand instrument has been the predominant method used for the dissection and/or collection of samples in many studies [2–5,7–11,23–26]. The design of this instrument severely limits precision because of its propensity to remove softer dentin in clumps and its inaccuracy in removal of the firmer dentin located beyond clinical excavations based on tactile perception. However, researchers using fluorescent *in situ* hybridization and transmission electron microscope methods report that taxa are present, viable and of high interest in this area [25,27]. In addition, smaller lesions such as pit and fissure lesions present challenges to spoon excavator use because the instrument does not fit into the lesion, blocks visual access, and does not remove the more firm carious tissue accurately. The new method uses high-quality micro-size carbide burs, which are new and sterile for each dissected layer. Importantly, the micro-size bur can access all types and sizes of lesions, precisely dissect soft or firm dentin and penetrate pits and fissures in enamel. The sterile BHI medium-dampened micro-size sterile brushes efficiently harvest the fine carious fragments created by the micro-size bur. The very small fragment size eliminates the problem of dentin clumps and the need for homogenization with its possibilities of damage to the microbes and microbe retention within larger pieces. In addition, the micro-size instruments used in conjunction with the surgical microscope provide the vision and access necessary for aseptic withdrawal of dissected samples without inadvertent contact with surrounding hard or soft tissues, biofilms or saliva. The incorporation of contaminants from these sources can result in the overestimation of microbial abundance that may not be recognized easily by taxa identity because of the similarity of the genera immediately surrounding and within a lesion [8].

Comparing the taxa reported in the 14 layered lesion diagrams in Figure 6 to taxa reported in other studies, we reviewed studies conducted over a 15-plus year period from various parts of the world and selected five studies that enumerated the microbes identified and reported the most predominant genera found [2,4,23,24,28]. A key point that must be kept in mind is that our 14 lesions were selected to test the method itself for robustness, and not as a test of the lesions. Hence, the 14 lesions display variables were deliberately unrestricted and include differences in lesion type, tooth type, lesion severity or stage and lesions that were untreated and treated, both operatively and non-operatively. Taking this caveat into consideration, we found the following six genera were predominant across our 14 lesions, from their superficial through their deepest dissected layer: *Actinomyces* (100%), *Streptococcus* (100%), *Rothia* (86%), *Propionibacterium* (71%), *Veillonella* (64%) and *Lactobacillus* (43%). Despite many differences between the five studies to which we compared our most predominant taxa, such as type of lesion, age of subjects, and taxa harvest and identification methods, all five reported three to five of these same six genera among their most predominant. Across our 14 example lesions, *Streptococcus mutans* was found in 38% of the untreated lesions and in 67% of the treated lesions. Well-known studies using culture [29] and later next-generation sequencing (NGS) [8] show that *Streptococcus mutans* is not present in all carious lesions.

Comparing the taxa within the layers of our 14 example diagrams to other studies is challenging owing to the uniqueness of the shallow-layer harvest and data display method. Although a number of studies [6,21,23,25,26,28,30], dating as far back as the early 1900s [21], describe dividing their test lesions into segments or layers that varied in number from two to five, none sequenced and reassembled the layers sequentially to show the pre-harvest positions of the taxa relative to lesion depth. However, two studies [23,30] that divided the dentin in their lesions into two layers (superficial dentin and deep dentin) reported the taxa identified in each layer separately. A comparison of our method with these two studies was attempted despite significant differences in the methods used and the limited sample sizes (see Table 2). Although the three studies differ in methodologies, it is of interest to compare genera frequencies as if the same methodology had been used. The most direct comparisons can be made using the genera observed in all three studies (*Actinomyces, Lactobacillus, Propionibacterium*). If the confidence intervals of two estimated frequencies overlap, the conclusion is that there is no statistical evidence of a difference in the observed estimates. Using this method, there appear to be no statistically significant differences in those genera for which comparisons can be made. However, because of the small number of teeth in each study, the confidence intervals of the estimated frequencies are wide, meaning that there is little statistical power in these comparisons. To obtain valid comparisons of layered harvesting, further research that includes larger sample sizes is needed.

**Table 2.** Comparison of the shallow-layer harvest and data display method with two other studies that harvested dentin in two layers (shallow dentin and deep dentin) and reported genera identified separately in each layer. The table reports genera as percent of teeth where each specific genus was observed in each layer (frequency percent). Confidence intervals are listed under each frequency percent reported.

Genera (alphabetical listing)	Hoshino et al. 1985 [30] (3 teeth) (ID by biochemicals & morphology)		Shallow-Layer Harvest & Data Display Method (7 of the 8 untreated lesions) (ID by culture & Sanger)		Liu et al. 2020 [23] (8 teeth) (ID by Illumina MiSeq)	
	Shallow dentin	Deep dentin	Shallow dentin	Deep dentin	Shallow dentin	Deep dentin
						
**Actinomyces**	**67%** (95% CI: 10, 99)	**100%** (95% CI: 30,100)	**100%** (95% CI: 60,100)	**57%** (95% CI: 20, 89)	**63%** (95% CI: 26, 91)	**63%** (95% CI: 26, 91)
**Arachnia**	**67%** (95% CI: 10, 99)	**67%** (95% CI: 10, 99)				
**Bifidobacterium**	**67%** (95% CI: 10, 99)	**33%** (95% CI: 1, 90)				
**Clostridiuim**	**67%** (95% CI: 10, 99)	**33%** (95% CI: 1, 90)				
**Corynebacterium**			**14%** (95% CI: 1, 57)	**14%** (95% CI: 1, 57)	**50%** (95% CI: 17, 83)	**63%** (95% CI: 26, 91)
**Eubacterium**	**100%** (95% CI: 30, 100)	**67%** (95% CI: 10, 99)				
**Lactobacillus**	**100%** (95% CI: 30,100)	**100%** (95% CI: 30,100)	**43%** (95% CI: 11, 80)	**14%** (95% CI: 1, 57)	**75%** (95% CI: 37, 96)	**75%** (95% CI: 37, 96)
**Leptotrichia**					**63%** (95% CI: 26, 91)	**38%** (95% CI: 9, 74)
**Olsenella**					**75%** (95% CI: 37, 96)	**88%** (95% CI: 49, 99)
**Prevotella**					**63%** (95% CI: 26, 91)	**63%** (95% CI: 26, 91)
**Propionibacterium**	**100%** (95% CI: 30,100)	**100%** (95% CI: 30,100)	**43%** (95% CI: 11, 80)		**75%** (95% CI: 37, 96)	**75%** (95% CI: 37, 96)
**Rothia**		**71%** (95% CI: 30, 96)				
**Selenomonas**					**75%** (95% CI: 37, 96)	**75%** (95% CI: 37, 96)
**Streptococcus**			**100%** (95% CI: 60,100)	**43%** (95% CI: 11, 80)	**50%** (95% CI: 17, 83)	**50%** (95% CI: 17, 83)

Note: All three studies reported *Actinomyces*, *Lactobacillus* and *Propionibacterium* present.

Culture with Sanger sequencing is currently used with this new harvest method. All the data in this report reflect culture with Sanger sequencing. The inability to culture all microbes is accepted as an innate disadvantage in order to gain proof of microbe viability at the time of harvest through culture and the ability to estimate microbe absolute abundance per milligram and relative abundance by percent in each layer. We are now learning culture-free NGS and spike-in methods [31–36] using layer sample aliquots to calculate the absolute abundance of microbes per milligram using NGS methods. More sensitive discrimination of taxa is a major advantage of NGS. However, generally, NGS methods do not confirm microbe viability and currently do not report microbe absolute abundance. Since neither culture nor non-culture methods alone provide all the information, in future work, we plan to use both methods in tandem to take advantage of the current benefits of each. Use of both methods in tandem is a technique recognized as helpful by others [2,37,38]. The shallow-layer harvest and data display method described in this report adapts equally well to both culture-dependent and culture-independent sequencing methods.

Limitations of the method include its dependence on a team and non-fluctuating performance of commercial products, such as the quality of the fragment collection brushes and the performance of the anaerobic chambers during lesion dissection. Both limitations require constant vigilance. Additionally, the size of the team could be a limitation. However, team size depends on administrative preference. We use eight team members to provide immediate processing of the samples, as they are generated to avoid possible harm by freezing the microbes we intend to culture. Access to equipment such as a surgical microscope, microbalance, or anaerobe chamber(s) may be another limitation. However, these components are vital to the method’s design and purposes.

In summary, the use of this new shallow-layer microbe harvest and data display method revealed the following new information about dental caries lesions: (1) Taxa pre-harvest spatial location relative to the lesion pulpal depth; (2) Pre-harvest transitions and fluctuations in taxa diversity and abundance (absolute and relative) throughout the lesion; and (3) Location of the advancing front along with identification of the taxa and their absolute and relative abundance at this critical site. This new detail and perspective on the taxa within dental caries lesions could be helpful in studying the functional genomics of dental caries, in the development of diagnostic instruments, and in validating new non-operative and operative treatments designed to halt dental caries progression.

## Supplementary Material

Supplementary material Supplementary material Figure S1.

Supplementary material Supplementary material Table S1.

## Data Availability

Sequencing data in this study are publicly available in Zenodo at https://doi.org/10.5281/zenodo.17601960. The supporting data include metadata, phylogenetic information with final identifications and consensus sequences.
