# Supplementary material for: Dental caries shallow-layer microbe harvest and data display method shows taxa pre-harvest spatial positions, absolute and relative abundance and diversity related to lesion pulpal depth
Source: J Oral Microbiol. 2025 Dec 4;17(1):2593129. doi: 10.1080/20002297.2025.2593129 (PMC12679847; doi:10.1080/20002297.2025.2593129)

**SUPPLEMENTARY MATERIAL:**

Manuscript Title: Dental caries shallow-layer microbe harvest and data display method shows taxa pre-harvest spatial positions, absolute and relative abundance and diversity related to lesion pulpal depth

Figure S1: Serial dilution scheme and CFU/mg calculation


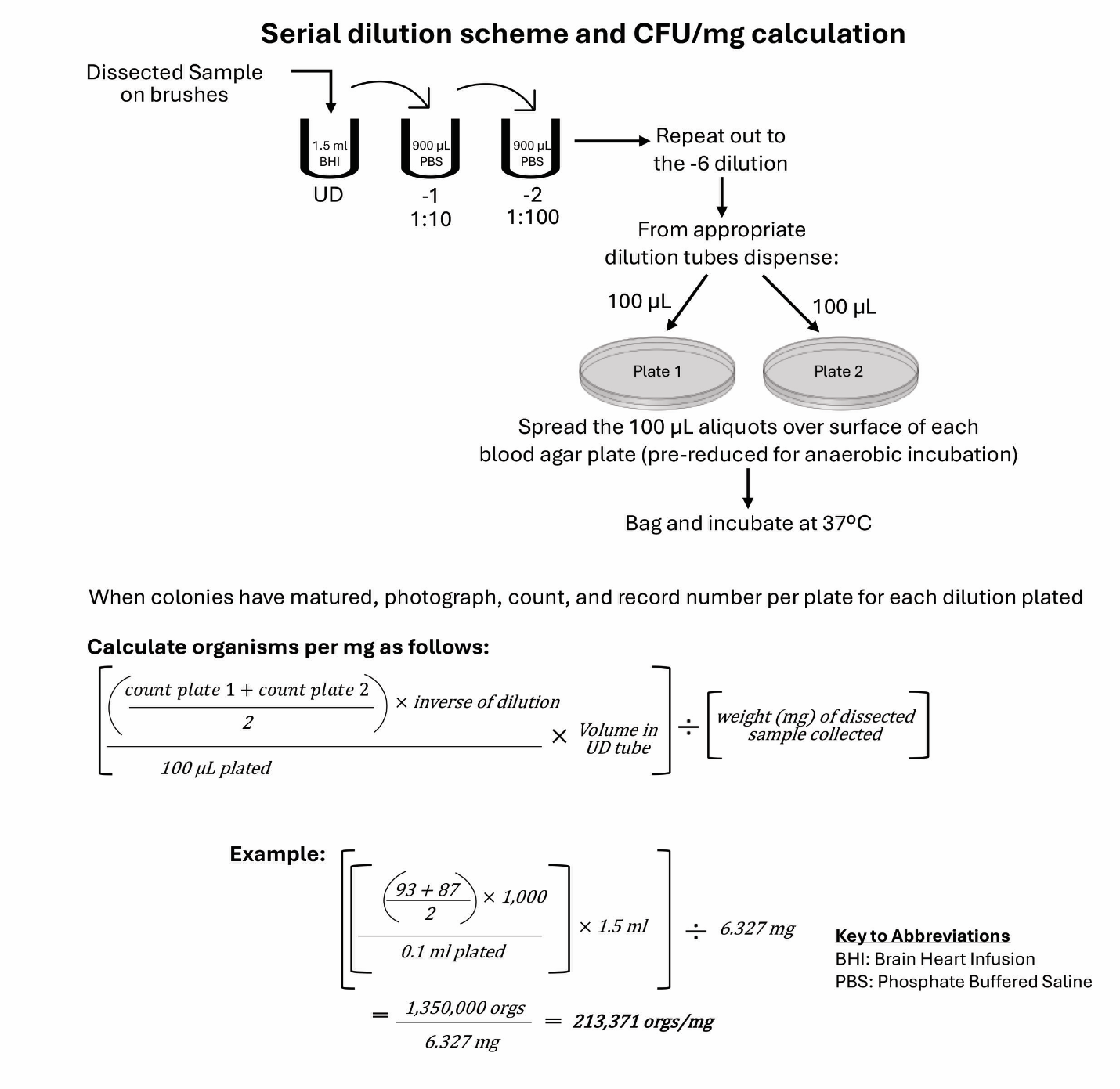

Supplement: Supplementary material — Figure S1. [file ZJOM_A_2593129_SM5853.docx]
